# The Practitioners' Bookshelf

**Published:** 1892-12-10

**Authors:** 


### THE PRACTITIONERS' BOOKSHELF.

### "A HISTORY OF MEDICAL EDUCATION." *

The work is what it professes to be, a history of medical education from the most remote times, when the practitioner was taught, as Hippocrates says, "by necessity," to the present scientific age, when his studies are supervised by paternal governments or a grandmotherly medical council. But it is something more than it professes to be, for it also contains an interesting, though necessarily incomplete, account of the general development of the healing art, and, with the exception of the American translation of Baas' "History of Medicine and the Medical Profession," is probably the best work on the subject now accessible to English readers. Books on medical history have usually been written on one of two principles. They consist either of a series of biographies of eminent physicians or heroes of medicine, enlivened with "stories of Abernethy" and other anecdotes, frequently more amusing than authentic, or they comprise critical accounts of the various medical systems and doctrines which have succeeded one another through the ages, together with epitomes and abstracts of the surviving works in which those doctrines are most clearly formulated. The first is the plan generally adopted by the few English writers who have attempted the subject in recent times, and we may find examples of the second in the laborious French and German treatises of Bouchut, Haeser, and Daremberg. Dr. Puschmann has dis-

* "A History of Medical Education," from the most remote to the most recent times. By Dr. Theodor Puschmann. Translated by Evan H. Hare, M.A., F.R.C.S. (H. K. Lewis.)

covered a third and very attractive mode of giving a historical outline of the healing art, namely, by describing how medical practitioners taught their pupils at various periods, and what they were able to teach them. He does not, however, appear to have intended to write a "History of Medicine," a form of literature with which the countrymen of Sprengel, Hecker, Baas, and Haeser are already amply provided; and this explains the occurrence of important omissions which prevent us from considering the present work an entirely satisfactory remedy for our own poverty in that respect. Thus, to take a single example, though we find a short biography of Galen followed by a sketch of his anatomical and physiological knowledge, no attempt is made to describe his medical system, without which a history of medicine somewhat resembles the play of Hamlet minus the hero. In periods of which comparatively little is known, as, for instance, the pre-Hippocratic and early mediæval epochs, Dr. Puschmann is able to include in his work almost all the historical facts which are worth recording, and such subjects as the medicine of the Talmudists, and the Arabic hospitals are more thoroughly discussed than in any of the larger medical histories above mentioned. But in such busy times as the Hippocratic, Galenic, and post-Harveian ages, he naturally breaks down under the weight of his material and is obliged to confine himself almost entirely to his special subject. It would be unfair, however, to find fault with a work for omitting what it does not pretend to give, and, considered simply as a history of medical education, the volume before us deserves the highest praise.

We cannot here follow the learned professor in his long and interesting course, but the two following extracts may give some slight idea both of the scope of the work and the style of the translation. Speaking of the medicine of the Talmudists, the author shows that they made extensive and successful use of experiments on animals; for "in this way they found that wounds of the kidney are not always fatal, and that the spleen can be removed, and even the uterus cut out without the death of the animal resulting." Scholars in the Greek schools of the fourth century seem to have somewhat resembled the traditional medical student of forty years ago, and we are told that on one occasion some of them tossed an unpopular tutor in a blanket till he fainted. They were thus rebuked by the sophist Libanius: "Bad enough were it for students to lay hands on an ordinary citizen, to insult a goldsmith, to provoke a shoemaker, to beat a carpenter, to kick a weaver, to maul a shopkeeper, to threaten an oil merchant, but to ill-use a pedagogue is an injury to one occupying a most respectable and useful position, and deserves chastisement with the rod and the whip."

In so extensive a work some errors will always be discoverable, but the few we have noticed are of little importance. It is hard upon Erasistratus that medical historians should persistently copy from one another the assertion that he thought the motor nerves originate from the membranes of the brain, for even his enemy, Galen, tells us that he only held this view in his younger days, and afterwards corrected the mistake by careful dissections. The statements that Bernard of Gordon was a Scotchman, that Canani first noticed valves in the veins, and that Christian lunatic asylums originated in Spain, are also open to question. As in most German scientific works, almost every statement is supported by references to original authorities, which will probably be of much assistance to future medical historians. But even Dr. Puschmann seems sometimes to forget that the first rule of historic composition is, "Verify your references." Thus, on page 174, we are told that in the Damascus hospital, "Such admirable care was taken of the sick that many, as Abdallatif states, feigned sickness so as to be allowed to remain there." But on referring to the authority mentioned we find that this statement is not made by Abdallatif, but by another writer who lived two centuries later, and that he does not say that many persons feigned sickness, but only that a certain friend of his did so.

The latter part of the book is occupied by an account of the present state of medical education in the various European countries and in North America, and it concludes with some very interesting "final considerations," in which Dr. Puschmann describes what he considers an ideal course of study. Among other things he holds that it is of the utmost consequence that the preliminary education of a medical student should be in no way inferior to that required for the other learned professions, but he objects to the present school routine, that while it stores the memory, exercises the understanding, and develops the thinking faculties, it forgets to rouse the powers of observation and to sharpen the senses. We cannot help thinking that, had this work appeared in two separate parts, one comprising a general history of medicine, written on the same lines, but somewhat more complete, the other strictly confined to the subject of medical education, they would probably have appealed to a larger circle, and have been more widely read than is likely to be the case with the present handsome and interesting but rather bulky and expensive volume.

Mr. Hare has done his work well, and has produced a fluent and readable English version, but his careful accuracy sometimes allows a German idiom to show through, and prevents our forgetting that the book is a translation. His editorial notes are confined to the section on English medical education at the present day, which he has brought thoroughly up to date, and even slightly beyond it, for the foundation of the Albert University is described as an accomplished fact. One small alteration, however, is still necessary, for it is now nearly ten years since candidates for the Oxford degree were required to translate passages from Galen or Hippocrates. A few other slight errors might be noted for future correction. On page 32, for instance, we read that, among the Parsees, "the healing art stood in intimate relation to culture," where the German "cultus" should evidently be rendered "religion" or "theology." On page 79 it is said that Erasistratus distinguished the motor from the sensory nerves; but later on (p. 101) we find that "Rufus the Ephesian was the first to bring to light the distinction of nerves into motor and sensory," an apparent contradiction which might be avoided by giving "hervorgehoben" its full sense of "emphasised" or "made prominent." On page 384 there is a very curious slip, for the translator makes Dr. Puschmann accuse Swift of marrying two brides, whereas in the German the accusation is that he married neither of them (nicht zwei Bräute betrogen haben, wie Swift). But these slight imperfections, like those noticed above, in no way detract from the value and interest of a book which may be fairly recommended as a most excellent and complete "History of Medical Education," and as the best substitute for a "History of Medicine," which we at present possess.

